# Exposure to HIV-protease inhibitors selects for increased expression of P-glycoprotein (ABCB1) in Kaposi's sarcoma cells

**DOI:** 10.1038/bjc.2011.275

**Published:** 2011-08-09

**Authors:** M B Lucia, R Anu, M Handley, J-P Gillet, C-P Wu, G M De Donatis, R Cauda, M M Gottesman

**Affiliations:** 1 Laboratory of Cell Biology, Center for Cancer Research, National Cancer Institute, NIH, 37 Convent Drive, Room 2108, Bethesda, MD 20892, USA; 2 Department of Infectious Diseases, Catholic University, Rome, Italy

**Keywords:** P-glycoprotein, ABCB1, Kaposi's sarcoma, HIV-protease inhibitors, multidrug resistance

## Abstract

**Background:**

Given that HIV-protease inhibitors (HIV-PIs) are substrates/inhibitors of the multidrug transporter ABCB1, can induce ABCB1 expression, and are used in combination with doxorubicin for AIDS-Kaposi's Sarcoma (KS) treatment, the role that ABCB1 plays in mediating multidrug resistance (MDR) in a fully transformed KS cell line (SLK) was explored.

**Methods:**

The KS cells were exposed to both acute and chronic treatments of physiological concentrations of different HIV-PIs (indinavir, nelfinavir, atazanavir, ritonavir, or lopinavir), alone or together with doxorubicin. The ABCB1 mRNA and protein expression levels were then assessed by qRT–PCR and western blotting, flow cytometry, and immunofluorescence.

**Results:**

Chronic treatment of SLK cells with one of the five HIV-PIs alone or together resulted in increased resistance to doxorubicin. Co-treatment with one of the HIV-PIs in combination with doxorubicin resulted in a synergistic increase in resistance to doxorubicin, and the degree of resistance was found to correlate with the expression of ABCB1. The SLK cells were also revealed to be cross-resistant to the structurally unrelated drug paclitaxel.

**Conclusion:**

These studies suggest that ABCB1 is primarily responsible for mediating MDR in SLK cells selected with either HIV-PIs alone or in combination with doxorubicin. Therefore, the roles that ABCB1 and drug cocktails play in mediating MDR in KS *in vivo* should be evaluated.

Despite recent advances in the treatment of cancer, drug resistance is still the major cause of anticancer chemotherapy failure in the clinic. Resistance manifests itself as an initial lack of a significant response to treatment or regrowth of a tumour after an initial response. Under these circumstances, tumour cells frequently exhibit simultaneous resistance to multiple structurally unrelated anticancer drugs, a phenotype known as multidrug resistance (MDR) (Gottesman *et al*, 2002). A major cause of the MDR phenotype is the overexpression in tumour cells of members of a highly conserved family of transmembrane proteins characterised by an ATP-binding cassette (ABC), that is, the ABC superfamily of transporters (Gottesman *et al*, 2002). The ABC transporters efflux structurally unrelated compounds in an ATP-dependent manner, lowering the intracellular drug concentration (Gottesman *et al*, 2002). The paradigm for membrane-based mechanisms of cytotoxic drug resistance is P-glycoprotein (P-gp/ABCB1) selected by the *MDR1* gene that functions to efflux natural cytotoxic compounds such as anthracyclines, vinca alkaloids, and taxanes from the cells (Gottesman *et al*, 2002). However, at least 11 other transporters have been shown to cause resistance to at least one agent (Szakacs *et al*, 2006). Modulators of the function of ABC transporters have been used in the attempt to overcome MDR (Gottesman *et al*, 2002).

Kaposi's sarcoma (KS), a multifocal tumour of endothelial origin, remains the second most frequent tumour in HIV-infected patients worldwide. Although its incidence has significantly decreased in the highly active antiretroviral therapy (HAART) era (Boshoff and Weiss, 2002), it has become the most common cancer in Sub-Saharan Africa (Sasco *et al*, 2010). For advanced AIDS-related KS with extensive cutaneous and/or visceral manifestations, systemic chemotherapy in combination with HAART is indicated (Bower *et al*, 2008). However, despite widespread availability of these therapies, a long-lasting and complete remission is achieved in only half the patients (Nguyen *et al*, 2008). For patients with refractory or recurrent AIDS-KS, treatment algorithms are less well defined. However, taxanes are currently recommended in patients who had previously received anthracyclines (Dhillon *et al*, 2005; Bower *et al*, 2008).

The KS aetiology as well as current drug regimens for AIDS (HAART) and AIDS-KS (anthracyclines) may induce and/or select for a MDR phenotype through altered expression/function of ABC transporters. The KS cells are endothelial-derived cells infected with and transformed by Kaposi's sarcoma-associated herpesvirus (KSHV) (Dittmer and Krown, 2007). The KS spindle cells express MRP1 and ABCB1. However, the mechanism by which these proteins are expressed (i.e., induction *vs* selection) and whether these proteins play a role in mediating drug resistance is not known (Schwartsmann *et al*, 1989; Gupta *et al*, 1998). The HIV-protease inhibitors (HIV-PIs) are substrates/inhibitors of the ABC transporters ABCB1, ABCG2, ABCC1, and ABCC2 (Lee *et al*, 1998); induce and/or select for ABCB1 in human tumour cells (Perloff *et al*, 2000; Vishnuvardhan *et al*, 2003); and sensitise cells to chemotherapeutics such as vincristine, vinblastine, and doxycycline in an HIV-PI-dependent manner. Furthermore, anthracyclines are substrates of many of the ABC transporters (such as ABCB1, ABCG2, ABCC1, and ABCC2) and both acute and chronic treatment with anthacyclines alone can induce ABCB1 expression resulting in an MDR phenotype (Gottesman *et al*, 2002). Previously, Dupuis *et al* (2002) demonstrated that vinblastine-selected MDR CEM cells treated with HIV-PIs (including ritonavir and saquinavir) sensitised the cells to chemotherapeutics most likely by acting as inhibitors for ABCB1. They found that co-treatment of CEM cells with ritonavir or saquinavir and a chemotherapeutic (vinblastine, vincristine, or doxorubicin) potentiated chemotherapeutic cytotoxicity, and that saquinavir can induce ABCB1 expression in CEM cells (Dupuis *et al*, 2002, 2003a, 2003b). However, acute and chronic treatment with each of the HIV-PIs alone or the combination of HIV-PIs with anthracyclines, the current drug regimen for AIDS-KS, and their roles in an ABCB1-dependent MDR phenotype have never been explored.

Although advanced AIDS-KS may show partial response or regrowth (Evans *et al*, 2002), induction/selection of MDR in KS-derived cells has not yet been examined, although KS spindle cells express the ABC transporters (Schwartsmann *et al*, 1989; Gupta *et al*, 1998) and the individual drugs alone from the drug regimen for AIDS-KS (HIV-PIs and anthracyclines) can induce or select cells that express ABCB1. In the present study, we verified the selectability of the MDR phenotype in a fully transformed KS-derived cell line following prolonged exposure to doxorubicin. Furthermore, KS cells were exposed to both acute and chronic treatments of physiological concentrations of different HIV-PIs (indinavir, nelfinavir, atazanavir, ritonavir, or lopinavir) alone or together with doxorubicin.

## Materials and methods

### Drugs and chemicals

The HIV-PIs indinavir sulphate, nelfinavir, ritonavir, atazanavir sulphate, and lopinavir were obtained through the NIH AIDS Research and Reference Program, Division of AIDS, NIAID, NIH (Bethesda, MD, USA). Further quantities of indinavir sulphate and ritonavir were kindly provided by Merck (West Point, PA, USA) and Abbott Labs (North Chicago, IL, USA). The HIV-PIs were dissolved in dimethyl sulphoxide (DMSO – maximum content 0.5% v/v, a concentration that was found not to influence the results of the assays) with the exception of indinavir, soluble in aqua bidest. Rhodamine 123 (Rh123), MTT (3-4,5-dimethylthiazol-2-yl-2,5-diphenyl tetrazolium bromide), doxorubicin hydrochloride, and paclitaxel were purchased from Sigma Aldrich (St Louis, MO, USA) whereas Cyclosporine A was from Calbiochem (EMD Chemicals, Gibbstown, NJ, USA).

### Cells and culture conditions

The KS tumour-derived cell line SLK (Herndier *et al*, 1994) was provided by the NIH AIDS Research and Reference Program, Division of AIDS, NIAID, NIH. Cells were cultured in RPMI-1640 (Gibco BRL, Gran Island, NY, USA) containing 10% fetal calf serum (FCS), 1% L-glutamine, and 1% penicillin–streptomycin in humidified atmosphere with 5% CO_2_ at 37 °C. This cell line, similar to KS, is fully transformed and tumourigenic in mice but has lost the KSHV genome in culture (O’Hara *et al*, 2009). Doxorubicin and HIV-PI-resistant SLK subclones were selected from the original drug-sensitive parent SLK cell line by exposure to increasing concentrations of the respective drug. Accordingly, four sublines designated as SLK-D0.02, -D0.04, -D0.09 and -D2 were developed by exposing the parental cells to 0.02, 0.04, 0.09, or 2 *μ*M doxorubicin, respectively. The SLK cells were also challenged with five different HIV-PIs, indinavir sulphate (IDV), nelfinavir (NFV), ritonavir (RTV), atazanvir sulphate (ATV), and lopinavir (LPV). All of the HIV-PIs chosen are documented substrates/inhibitors of ABC transporters (Lee *et al*, 1998; Srinivas *et al*, 1998; Vishnuvardhan *et al*, 2003). The corresponding SLK-PI sublines were obtained by incubating cells with indinavir at concentrations of 10, 30, or 50 *μ*M, resulting in IDV10, IDV30, and IDV50, respectively; nelfinavir at concentrations of 2, 5, and 10 *μ*M, resulting in NFV2, NFV5, and NFV10, respectively; 1, 5, and 10 *μ*M atazanavir resulting in ATV1, ATV5, and ATV10, respectively; 1, 2.5, 5, 10, or 20 *μ*M ritonavir resulting in RTV1, RTV2.5, RTV5, RTV10, or RTV20, respectively; 1 and 10 *μ*M lopinavir resulting in LPV1 and LPV10, respectively; and 10 *μ*M lopinavir plus 2.5 *μ*M ritonavir resulting in LPV10/RTV2.5. Three SLK sublines were obtained through selection with 0.02 *μ*M doxorubicin plus 5 *μ*M of indinavir, nelfinavir, atazanavir, or ritonavir and named D/IDV5, D/NFV5, D/ATV5, and D/RTV5, respectively. In addition, a subline named D/LPV10/RTV5 was selected through exposure to doxorubicin 0.02 *μ*M and 10 and 5 *μ*M of lopinavir and ritonavir, respectively. The SLK cells were exposed to step-wise increasing concentrations of doxorubicin and HIV-PIs for 8 and 6 months, respectively. At 3 days before and during experiments, all selective drugs were omitted. The human epidermoid carcinoma, KB-3-1, and a KB-3-1 variant that overexpresses ABCB1, KB-V1, were cultured in Dulbecco's modified Eagle's medium (DMEM; Gibco) supplemented with 10% FCS, 1% L-glutamine, and 1% penicillin–streptomycin and were used as negative and positive controls for typical *MDR1-*expressing cells.

### Western blot analysis

For membrane protein extraction, the cells were lysed in ice-cold radioimmunoprecipitation assay buffer (RIPA) containing 2% Triton X-100, 40 mM Tris (pH 7.2), 300 mM NaCl, 10 mM EDTA, 500 mM NaF, and 100 mM Na_3_Vo_4_ supplemented with Complete Protease Inhibitors Cocktail (Sigma-Aldrich). Lysates were cleared by centrifugation at 4 °C, solubilised in 5 × SDS supplemented with 1 mM DTT, and total protein concentration was determined by a bicinchoninic acid protein assay (Pierce, Rockford, IL, USA) with bovine serum albumin (BSA) as standard. Proteins (50 *μ*g) were resolved on Nu-Page 3–8% Tris acetate gels (Life Technologies, Invitrogen, Carlsbad, CA, USA) and transferred to nitrocellulose membrane (Bio-Rad, Hercules CA, USA). Blots were blocked for nonspecific binding in Tris-buffered saline (TTBS) buffer containing 15 mM Tris, 154 mM NaCl, 0.1% Triton X-100, and 5% goat serum (Invitrogen). Membranes were incubated overnight at room temperature with the primary antibody against ABCB1 (C219, 1 : 1000) diluted in TTBS buffer plus 5% goat serum. Blots were then probed with anti-glyceraldehyde-3-phosphate dehydrogenase (GAPDH, 1 : 800 000; Applied Biosystems, Foster City, CA, USA). Membranes were washed in TTBS and incubated with an anti-mouse horseradish peroxidase-conjugated secondary antibody (1 : 10 000; Pierce). After the blots were washed with TTBS, immunoreactive bands were visualised using the enhanced chemiluminescence detection kit (ECL, Perkin Elmer, Waltham, MA, USA) and exposed to autoradiographic film (Kodak, purchased from Sigma Aldrich). The intensity of the bands was quantified using ImageQuant TL software (GE Healthcare Biosciences, Pittsburgh, PA, USA). The protein level of ABCB1 was normalised relative to the protein level of GAPDH.

### Flow cytometry

Expression of surface ABCB1 was detected by flow cytometry using the MRK-16 antibody as previously reported (Aleman *et al*, 2003), with slight modifications. Per sample, 10 000 events were collected and analysed on a FACSort flow cytometer equipped with Cell Quest software (Becton Dickinson, Franklin Lakes, NJ, USA). TheRh123 efflux assay was used to assess the functional activity of ABCB1 in the presence or absence of the inhibitor cyclosporine A (CsA) (Foxwell *et al*, 1989). Briefly, cells (5 × 10^5^) were incubated at 37 °C in the dark for 45 min in 4 ml of Iscove's modified Dulbecco's medium (IMDM) supplemented with 5% FCS containing Rh123 (0.5 *μ*g ml^–1^), and washed and resuspended in IMDM 5% FCS without Rh123 for an additional 45 min. Cells were then washed and immediately analysed for intracellular Rh123 fluorescence on a FACSort flow cytometer. A minimum of 10 000 events were collected for all samples.

### Immunofluorescence

Cells were fixed with a 3.7% glyceraldehyde solution dissolved in phosphate-buffered saline (PBS) for 10 min at room temperature, followed by permeabilisation in PBS–0.5% Triton X-100. After blocking in 1% BSA in PBS, cells were washed in PBS and incubated with anti-ABCB1 MRK-16 antibody (1 : 500). After extensive washing, the cells were stained with goat anti-mouse conjugated with Alexa Fluor 488 (1 : 1000, Invitrogen), washed, and mounted with Vectashield (Vector Laboratories, Burlingame, CA, USA) mounting medium containing the DNA stain 4′,6-diamidino-2-phenylindole (DAPI). Cell preparations were observed under a confocal laser scanning fluorescence microscope (LSM510, Carl Zeiss, MicroImaging, Thornwood, NY, USA).

### Preparation of total RNA

Total RNA from SLK cell lines was prepared using an RNeasy Micro kit (Qiagen, Valencia, CA, USA) as per the manufacturer's instructions. RNA was quantitated using NanoDrop ND-1000 spectrophotometer (NanoDrop Technologies Inc., Wilmington, DE, USA). The integrity of the RNA samples was assessed using an Agilent 2100 Bioanalyser (Agilent Technologies, Foster City, CA, USA) and then stored at −80 °C.

### Reverse transcription

Synthesis of cDNA from 1 *μ*g total RNA in a 20 *μ*l reaction volume was carried out using the High-Capacity cDNA kit with RNAse inhibitor (Applied Biosystems) as per the manufacturer's instructions. The reverse transcription conditions were as follows: 10 min at 25 °C, 120 min at 37 °C, and 5 min at 85 °C. Following reverse transcription, cDNA was stored at 4 °C.

### Taqman-based qRT–PCR

Expression levels of the *ABCB1* (MDR1/P-gp) gene were measured using the Hs00184491_m1 assay (Applied Biosystems); 18S was used as a housekeeping gene, Hs99999901_s1. In addition, 100 ng of cDNA was mixed with 2 × Taqman Universal PCR Master Mix (Applied Biosystems), loaded in triplicate on a 96-well plate, and run on an ABI Prism 7900 HT Sequence Detection System (Applied Biosystems) as per the manufacturer's instructions. Expression data were analysed using RQ Manager software (Applied Biosystems).

### MTT cytotoxicity assays

Cells were plated into flat-bottom 96-well plate at 2 × 10^3^–5 × 10^3^ per well and incubated for 24 h at 37 °C in 5% CO_2_ before addition of various concentrations of chemotherapeutic agents to the medium and incubated for another 72 h. After removal of medium, MTT (0.5 mg ml^–1^) in IMDM growth medium was added to each well. The plates were incubated at 37 °C under 5% CO_2_ for 4 h. The MTT solution was then removed from the wells, and 100 *μ*l acidified ethanol solution was added to dissolve the formazan crystals. After 15 min, the absorbance at 560 nm with a reference wavelength of 630 nm was read with a SpectraMax 384 microplate reader (Molecular Devices, Sunnyvale, CA, USA). Dose-response curves were plotted and the concentrations that gave 50% inhibition of cell growth (IC_50_) were calculated. The fold resistances were calculated from the ratio of the IC_50_ of the drug-exposed cell line to the IC_50_ of the sensitive parental SLK cells (resistance factor (RF)). Survival of cells that were not incubated with drug was expressed as 100%. Triplicate wells were used for each treatment. Assays were performed at least three times.

### Statistical analysis

Data are presented as means±s.d. and were analysed using GraphPad Prism Version 5.0 and InStat Version 3.05 (GraphPad Software, San Diego, CA, USA). Differences were compared using Student's *t*-test. A *P*-value of ⩽0.05 was considered significant. Drug combinations were evaluated for their ability to promote resistance synergistically or additively using the Bliss Model of Independence (Fitzgerald *et al*, 2006). The model states that in case two drugs A and B contribute additively to the resistance, then their combined effect can be described by the formula: IC_50A+B_=IC_50A_+IC_50B_−IC_50A_ × IC_50B_ where the IC_50A_ and IC_50B_ are the half-maximal inhibitory concentrations for doxorubicin and paclitaxel in cells pretreated with the compound A or B, respectively. The expected values obtained were compared with the ones experimentally determined using Student's *t*-test with a significance level of *P*⩽0.05. Analysis was performed using Sigma Plot 11.0 (Systat Software, Inc., Chicago, IL, USA).

## Results

### Long-term exposure to doxorubicin and HIV-PIs results in the development of resistance in SLK cells

Because of the fact that multidrug-resistant KS cells in HIV patients receiving HAART were reported, we wanted to determine if treatment with doxorubicin or HIV-PI treatment alone could induce/select resistant KS cells that exhibited a multidrug-resistant phenotype *in vitro.* Therefore, dose-response curves to doxorubicin and paclitaxel for the parental cell lines as well as cell lines exposed to HIV-PI or doxorubicin for 6–8 months were generated using MTT assays and the IC_50_ for doxorubicin and paclitaxel for each cell line was determined.

Initially, the IC_50_ values for doxorubicin were determined in the parental as well as in four doxorubicin-resistant cell lines. The IC_50_ values obtained from the dose-response curves demonstrate that long-term incubation with doxorubicin led to a 2.4- to 12.5-fold increase in IC_50_ and that the fold increase in resistance correlated with the increase in the doxorubicin concentration used for treatments where D0.02<D0.04<D0.09<D0.2 (Table 1). The SLK-DOX cell lines selected with the lowest physiological concentrations of doxorubicin (0.02 and 0.04 *μ*M; Mou *et al*, 1997) were then challenged with a different MDR-related drug, paclitaxel, a taxane compound. A clear cross-resistance of 6.3- to 47.7-fold resistance was observed (Table 2).

The HIV-PI-treated SLK cell lines were also analysed for sensitivity to doxorubicin. As shown in Table 1, HIV-PIs select for a significant increase of drug resistance, with a resistance factor ranging from 1.4 to 3.4 compared with SLK parent cells. Resistance to doxorubicin in HIV-PI-treated cells revealed that RTV2.5<ATV1<NFV2<NFV5<IDV50 (Table 1). An overall increase in doxorubicin resistance was detected for almost all HIV-PI-selected SLK cell lines (Table 1).

Next, we determined whether HIV-PI-treated SLK cell lines show cross-resistance to paclitaxel. Resistance to this compound is even more significant than that observed for doxorubicin, because of the fact that almost all tested cell lines show significantly higher IC_50_ values compared with parental SLK cells, with a resistance factor ranging from 2.05 to 8.5. Resistance to paclitaxel in HIV-PI-treated cells revealed that ATV1<RTV5<NFV10<NFV2<NFV5<IDV50 (Table 2). Furthermore, nelfinavir-treated SLK cell lines were also challenged with the liposomal formulation of doxorubicin (Doxil). Also in this case, NFV5 cells were significantly more resistant compared with SLK parent cells (IC_50_: 3.626±0.558 *vs* 2.037±0.628 for NFV5 cells and SLK parent cells, respectively; *P*<0.05). Importantly, when resistant SLK cell lines were maintained for >20 days in culture in the absence of doxorubicin and HIV-PIs, the MDR phenotype remained stable.

### Long-term co-treatment with doxorubicin and HIV-PIs acts synergistically on SLK cell line sensitivity

Analysis of SLK cells co-exposed to doxorubicin and HIV-PIs suggests a synergistic effect between these two compounds (Table 1). Co-treated SLK cell lines show significantly increased IC_50_ values compared with either parental SLK cells or those exposed to the anthracycline alone (Table 1). The same trend is seen in resistance to paclitaxel except that ritonavir-treated cells are more resistant than atazanavir-treated cells (Table 2). The Bliss independence drug interaction analysis further supports the synergistic effect between doxorubicin and HIV-PIs (Tables 1 and 2). Taken together, these results suggest the following hierarchy of potency to select for resistance: D/LPV/RTV > D/RTV5 D/ATV5 > D/NFV5> D/IDV5.

### HIV-PI-selected SLK cells express ABCB1 mRNA and protein

**Figure 1:**
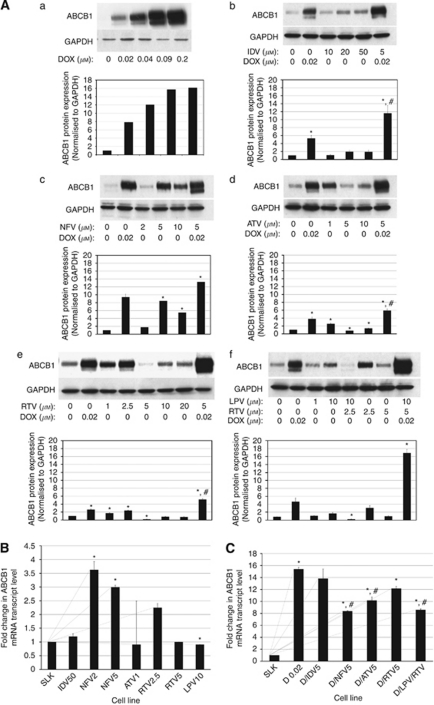
ABCB1 expression at the mRNA and protein levels in SLK cell lines selected with HIV-PIs alone or in combination with doxorubicin. (**A–C**) SLK cell lines treated with delivery group alone, various concentrations of doxorubicin (D) alone (a), various concentrations of either indinavir (IDV; b), nelfinavir (NFV; c), atazanavir (ATV; d), ritonavir (RTV; e), lopinavir (LPV; f), lopinavir/ritonavir (LPV/RTV; f), or co-treated with 0.02  *μ*M doxorubicin and the indicated HIV-PIs for 6–8 months. (**A**) Cells were harvested, lysed, resolved by SDS–PAGE gels, and analysed by western blotting with ABCB1 and GAPDH, the loading control. Bar graphs represent quantification of ABCB1 protein expression normalised to GAPDH. Data are presented as mean±s.e.m. Protein from KB-3-1 cells and ABCB1-overexpressing KB-V1 cells served as controls for specificity of the ABCB1 antibody. The analysis was repeated three times with protein extracts from separate cell lysates. (**B** and **C**) Cells were harvested and total RNA from SLK cells treated with various concentrations of HIV-PIs alone (**B**) or in combination with 0.02 *μ*M doxorubicin (**C**) was purified and analysed by qRT–PCR analysis with primers selective for ABCB1 and 18S, the control. Data represent transcript levels±s.d. normalised to ABCB1 mRNA transcript levels from SLK parental cells. ^*^P<0.05 *vs* SLK parent cells, ^#^*P*<0.05 *vs* D0.02.

Because of the fact that selection with HIV-PIs resulted in resistance to doxorubicin and paclitaxel, the two known substrates of ABCB1 (Gottesman *et al*, 2002), our next question was whether ABCB1 protein was upregulated in the doxorubicin- and taxol-resistant SLK cell lines. Immunoblot analysis of ABCB1 expression in doxorubicin-selected cells revealed a dose-dependent increase in the expression of ABCB1 (Figure 1Aa). Overall, HIV-PI treatment of SLK cells resulted in an inverse correlation between the levels of ABCB1 expression and the concentration of the HIV-PI used for selection. For example, selection of SLK cells with 1 *μ*M atazanavir resulted in a higher level of ABCB1 expression than selection with 5 or 10 *μ*M atazanavir (Figure 1Ad). The same basic trend was seen for all HIV-PIs, with the exceptions of indinavir (Figure 1Ab) and lopinavir (Figure 1Af). Selection of SLK cells with increasing physiological concentrations of lopinavir resulted in increased levels of ABCB1. The identity of the HIV-PIs was also important in determining the levels of ABCB1 protein expression where: NFV>RTV>ATV>LPV>IDV. These complex dose responses to HIV-PIs suggest a biphasic or multiphasic response of ABCB1 expression to HIV-PI treatment with inhibition of expression at higher HIV-PI levels.

Next, we determined the effect of combining either two HIV-PIs or co-treatment of doxorubicin and an HIV-PI on ABCB1 expression. The combination of lopinavir/ritonavir in a 4 : 1 ratio (Cvetkovic and Goa, 2003) resulted in a significant decrease of ABCB1 expression (Figure 1Af). SLK cell lines co-treated with doxorubicin and an HIV-PI showed higher levels of ABCB1 expression than with HIV-PI treatment alone. The synergistic increase in ABCB1 expression seen in cells co-treated with HIV-PIs and doxorubicin revealed that D/LPV/RTV>D/ATV5>D/RTV5>D/NFV5>D/IDV5 (Figure 1A). Not only did all the doxorubicin and HIV-PI-treated and co-treated resistant SLK cell lines express more ABCB1 than parental cell lines, but the levels of ABCB1 expressed correlated well with the levels of resistance (Figure 1A and Table 1). However, indinavir-selected SLK cell lines expressed the least amount of ABCB1, but were the most resistant to doxorubicin (Figure 1Ab and Table 1). These data suggest that indinavir-selected SLK cell lines may be doxorubicin-resistant in an ABCB1-independent manner.

Next, we determined whether upregulation of ABCB1 protein in response to HIV-PI treatment alone or co-treatment with HIV-PIs and doxorubicin resulted in altered ABCB1 mRNA levels. Total mRNA was purified and analysed by qRT–PCR for ABCB1 and the amount of ABCB1 mRNA transcript level was normalised to 18S mRNA expression. When ABCB1 mRNA levels of resistant HIV-PI-treated SLK cell lines were compared with the levels present in the parental SLK cell line, ABCB1 mRNA levels in NFV2>NFV5>RTV2.5>IDV50>ATV1 (Figure 1B). These levels did not correspond well to the degree of doxorubicin resistance found in the cytotoxicity assays. The levels of ABCB1 mRNA found in the doxorubicin and HIV-PI co-treated SLK cell lines revealed that D.02>D/IDV5>D/RTV5>D/ATV5>D/NFV5>D/LPV/RTV (Figure 1C). These results also did not correspond well with the degree of resistance to doxorubicin.

### SLK cell lines treated with HIV-PIs alone or in combination express functional ABCB1 at the cell surface

**Figure 2:**
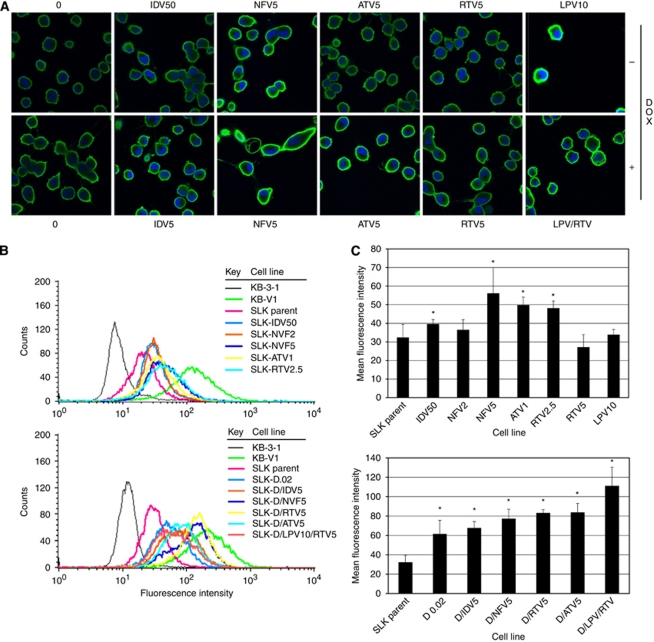
Localisation of ABCB1 expression in SLK cell lines selected with HIV-PIs alone or in combination with doxorubicin. (**A**) Confocal immunofluorescent images of ABCB1 expression in SLK cell lines treated with delivery group alone, 0.02 *μ*M doxorubicin (DOX) alone, 50 *μ*M indinavir (IDV50), 5 *μ*M nelfinavir (NFV5), 5 *μ*M atazanavir (ATV5), 5 *μ*M ritonavir (RTV5), 10 *μ*M lopinavir (LPV10), 10 *μ*M lopinavir plus 5 *μ*M ritonavir (LPV/RTV), or co-treated with 0.02 *μ*M doxorubicin and the indicated HIV-PIs for 6–8 months. (**B**) Histogram generated from a FACS analysis of the SLK parental cells selected with nothing (SLK parental), 50 *μ*M indinavir (IDV50), 2 *μ*M nelfinavir (NFV2), 5 *μ*M nelfinavir (NFV5), 1 *μ*M atazanavir (ATV1), 2.5 *μ*M ritonavir (RTV2.5), 5 *μ*M ritonavir (RTV5), or 10 *μ*M lopinavir (LPV10) incubated with anti-ABCB1 and visualised with Alexa Fluor 488 conjugated goat anti-mouse (upper panel). The lower panel is generated in the same fashion for SLK cells selected with 0.02 *μ*M doxorubicin alone (D0.02) or 5 *μ*M of indinavir (D/IDV5), nelfinavir (D/NFV5), atazanavir (D/ATV5), ritonavir (D/RTV5), or 10 *μ*M lopinavir plus 5 *μ*M ritonavir (D/LPV/RTV) in combination with 0.02 *μ*M doxorubicin. KB-3-1 cells and ABCB1-overexpressing KB-V1 cells were used as negative and positive controls for protein expression, respectively. (**C**) Bar graphs represent the mean±s.d. of ABCB1 expression from resistant HIV-PI-selected SLK cell lines (upper panel) and from resistant HIV-PI and doxorubicin co-selected SLK cell lines (lower panel) from three independent experiments. ^*^P<0.05.

Because of the fact that western blotting of whole cell lysates does not reveal protein location, we next investigated whether treatment with HIV-PIs alone or in combination with doxorubicin altered ABCB1 localisation in SLK cells using immunofluorescence and FACS. The SLK cells treated with HIV-PIs alone or in combination with doxorubicin were grown on coverslips, permeabilised, fixed, probed with MRK-16, an antibody that recognises the extracellular epitope of ABCB1, and visualised with a secondary antibody conjugated with Alexa Fluor 488. The confocal images revealed that ABCB1 was primarily localised to the plasma membrane and that ABCB1 expression profiles correlated well with western blotting expression profiles (Figure 2A).

Next, the expression of ABCB1 at the plasma membrane was quantified using FACS analysis. The ABCB1 expression on SLK cells treated with HIV-PIs alone or in combination with doxorubicin was probed with MRK16, visualised with a secondary antibody conjugated to Alexa Fluor 488, and analysed using FACS analysis. We observed a significant increase of surface ABCB1 expression, which correlated with the degree of doxorubicin resistance (Figure 2B and C) except in the case of ritonavir 2.5 (RTV2.5; Figure 2B and C). Therefore, either the activity of ABCB1 is greatly increased in these cells or there are ABCB1-independent mechanisms of resistance to doxorubicin.

**Figure 3:**
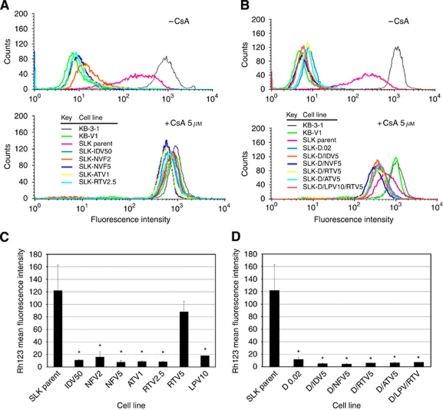
ABCB1-dependent efflux of rhodamine 123 (Rh123) in resistant SLK cells selected with HIV-PIs alone or in combination with doxorubicin. (**A**) Histograms represent FACS analysis of Rh123 absorbance in SLK cells selected with nothing (SLK parent), 50 *μ*M indinavir (IDV50), 2 *μ*M nelfinavir (NFV2), 5 *μ*M nelfinavir (NFV5), 1 *μ*M atazanavir (ATV1), 2.5 *μ*M ritonavir (RTV2.5), 5 *μ*M ritonavir (RTV5), or 10 *μ*M lopinavir (LPV10) after incubation in IMDM supplemented with 1.3 *μ*M Rh123 in the presence and absence of an ABCB1 selective inhibitor cyclosporine A (CsA) for 45 min and resuspension in IMDM supplemented without and with 5 *μ*M CsA for 45 min (upper and lower panel, respectively). (**B**) Lower panel was generated in the same fashion for SLK cells selected with 0.02 *μ*M doxorubicin alone (D0.02) or 5 *μ*M of indinavir (D/IDV5), nelfinavir (D/NFV5), atazanavir (D/ATV5), ritonavir (D/RTV5), or 10 *μ*M lopinavir plus 5 *μ*M ritonavir (D/LPV/RTV) in combination with 0.02 *μ*M doxorubicin. The KB-3-1 cells and ABCB1-overexpressing KB-V1 cells were used as negative and positive controls for ABCB1 protein function, respectively. (**C**) Bar graphs shows mean±s.d. of Rh123 efflux values from resistant HIV-PI-selected SLK cell lines and from resistant HIV-PIs and doxorubicin co-selected SLK cell lines (**D**). ^*^P<0.05.

Although ABCB1 is expressed at the cell surface, it may not be functional. Therefore, ABCB1 function was assessed with an ABCB1 fluorescent substrate, Rh123 (Lee *et al*, 1994). Efflux was measured in SLK cell lines treated with HIV-PIs alone or in combination with doxorubicin. Controls were the ABCB1-expressing KB-V1 cell line and its non-ABCB1-expressing parental KB-3-1 cell line. A significant increase of the efflux activity in all resistant SLK cell lines as well as KB-V1 cells was observed compared with parental cells (Figures 3A–D). A representative experiment showing Rh123 efflux by HIV-PI-selected SLK cell lines (Figure 3A, upper panel) and co-selected SLK cell lines (Figure 3B, upper panel) is shown. To confirm ABCB1-dependent efflux, CsA, an inhibitor of ABCB1 efflux function (Foxwell *et al*, 1989), was used. The Rh123 efflux was almost completely abolished by 5 *μ*M CsA in all resistant SLK cell lines as well as the ABCB1-expressing KB-V1 positive control cell line, as documented by the shift of the histograms towards higher levels of Rh123-related fluorescence (Figures 3A and B, lower panels), further suggesting that cells treated with HIV-PIs alone or in combination with doxorubicin express functional plasma membrane localised ABCB1.

### Short-term treatment shows that doxorubicin induces ABCB1 expression, whereas HIV-PI treatment selects for ABCB1 expression in SLK cells

**Figure 4:**
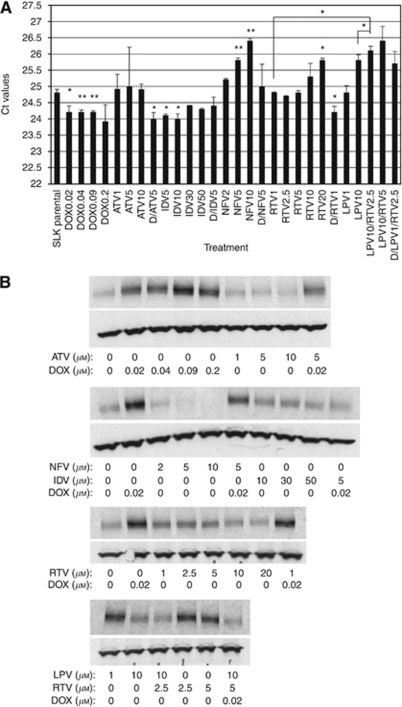
Whereas doxorubicin induces ABCB1 in SLK cells, HIV-PIs do not induce ABCB1 in SLK cells. The SLK cells were treated with nothing (SLK parent), 0.02, 0.04, 0.09, or 0.2 *μ*M doxorubicin (D0.02, D0.04, D0.09, or D0.2, respectively); 1, 5, or 10 *μ*M atazanavir (ATV1, ATV5, or ATV10, respectively); 5, 10, 30, or 50 *μ*M indinavir (IDV5, IDV10, IDV30, or IDV50, respectively); 2, 5, or 10 *μ*M nelfinavir (NFV2, NFV5, or NFV10, respectively); 1, 2.5, 5, 10, or 20 *μ*M ritonavir (RTV1, RTV2.5, RTV5, RTV10, or RTV20, respectively); 1 or 10 *μ*M lopinavir (LPV1 or LPV10, respectively); 10 *μ*M lopinavir and 2.5 *μ*M ritonavir (LPV10/RTV2.5); 10 *μ*M lopinavir and 5 *μ*M ritonavir (LPV10/RTV5); or 0.02 *μ*M doxorubicin plus either 5 *μ*M of atazanavir (D/ATV5), 5 *μ*M indinavir (D/IDV5), 5 *μ*M nelfinavir (D/NFV5), 1 *μ*M ritonavir (D/RTV1), or 1 *μ*M lopinavir plus 2.5 *μ*M ritonavir (D/LPV1/RTV2.5) for 72 h. (**A**) Bar graph of Ct values for ABCB1 mRNA normalised to 18S, the loading control, from SLK cells treated with various concentrations of HIV-PIs alone or in combination with doxorubicin. Cells were harvested, total mRNA was purified, ABCB1 message was amplified using qRT–PCR analysis with ABCB1 selective primers and normalised to 18S, the loading control. ^*^P<0.1, ^**^P<0.05. (**B**) Immunoblot analysis of lysates from SLK cells treated with nothing, various concentrations of doxorubicin alone, various concentrations of the five different HIV-PIs (atazanavir, indinavir, nelfinavir, ritonavir, or lopinavir) alone, various concentrations of combined HIV-PIs (ritonavir and lopinavir), doxorubicin and the different HIV-PIs, or doxorubicin plus ritonavir and lopinavir for 72 h. Cells were harvested, lysed, resolved by SDS–PAGE gels, and analysed by western blot analysis using antibodies selective for ABCB1 and GAPDH, the loading control.

Because of the fact that all treatment combinations resulted in ABCB1 expression, we next determined whether ABCB1 expression is a result of induction and/or selection. Therefore, SLK cells were incubated for 72 h in the presence of one of the HIV-PIs alone or in combination with doxorubicin. Expression profiles for ABCB1 mRNA and protein were determined using qRT–PCR and western blotting, respectively. Short-term treatment of SLK parental cells with increasing concentrations of doxorubicin alone resulted in a concentration-dependent increase in ABCB1 mRNA and protein (Figures 4A and B). Treatment with any one of the HIV-PIs alone did not alter ABCB1 expression, with the exception of cells treated with 5 *μ*M NFV, 10 *μ*M NFV, and 10 *μ*M LPV, which resulted in decreased levels of ABCB1 mRNA and protein compared with SLK parental cells (Figures 4A and B). Co-treatment of SLK cells with 10 *μ*M LPV plus 2.5 *μ*M RTV or 0.02 *μ*M doxorubicin plus 10 *μ*M LPV and 5 *μ*M RTV also resulted in decreased ABCB1 expression at the mRNA and protein levels (Figures 4A and B). Whereas co-treatment with doxorubicin plus an HIV-PI increased ABCB1 expression levels for each of the HIV-PIs, levels of ABCB1 did not differ from treatment with doxorubicin treatment alone (Figures 4A and B). Therefore, doxorubicin induces ABCB1 expression in SLK cells, whereas treatment with HIV-PIs alone selects for cells expressing ABCB1.

## Discussion

The present study shows that extended exposure to physiologically relevant concentrations of HIV-PIs selects for a stable MDR phenotype in a fully transformed KS-derived cell line. In addition, HIV-PIs act synergistically with the anthracycline doxorubicin to select for higher levels of ABCB1 (Figures 1A and 2). These observations may be of particular interest given the growing problem of cancer in the HIV-infected populations and the antitumour activity shown by HIV-PIs.

Despite a decrease in incidence, likely because of the reversal of immunosuppression that follows antiretroviral treatment initiation, KS remains a disease with a wide spectrum of severity. In particular, recurrence and/or disease progression despite chemotherapy and/or successful antiretroviral therapy are not uncommon (Maurer *et al*, 2007; Bower *et al*, 2008; Sullivan *et al*, 2009), suggesting that immune reconstitution alone may not be the unique mechanism able to influence KS clinical outcome (Pellet *et al*, 2001). Drug-transporting ABC transporters, including ABCB1, were found to be associated with acquired MDR in cancer cells. Although clinically suspected (Presant *et al*, 1993), the MDR phenomenon has never been fully characterised in KS spindle cells that have been found to express ABC transporters (Schwartsmann *et al*, 1989; Gupta *et al*, 1998). In light of this, we started by analysing the effect on SLK cells of long-term exposure to doxorubicin. Our results show that this compound is able to induce dose-dependent resistance together with a significant cross-resistance to the taxane compound paclitaxel, suggesting ABCB1-related resistance (Tables 1 and 2 and Figure 4). The increased expression of ABCB1 at the mRNA and protein levels, as well as its active functional status, strongly supports this hypothesis (Figure 1Aa and data not shown).

As KS-derived spindle cells are able to develop a stable MDR phenotype when challenged with common cytotoxic compounds, we next analysed whether HIV-PIs may act similarly. Five different HIV-PIs (i.e., IDV, NFV, ATV, RTV, and LPV) were chosen based on their known ability to act as substrates for ABC transporters (Lee *et al*, 1998; Srinivas *et al*, 1998; Vishnuvardhan *et al*, 2003) and their antitumour properties (Bernstein and Dennis, 2008; Chow *et al*, 2009). These compounds were tested either alone or together with doxorubicin at concentrations in line with those achieved in HIV-infected patients undergoing antiretroviral treatment. Our data clearly highlight the ability of low and completely physiological concentrations of HIV-PIs to select for an MDR phenotype through increased ABCB1 expression and function in KS-derived spindle cells (Figures 1, 2 and 3). Interestingly, the mechanisms resulting in ABCB1 expression of doxorubicin and HIV protease inhibitors are different. Whereas doxorubicin induced ABCB1 expression and functionality in SLK cells after acute treatment (72 h), ABCB1 was only expressed in SLK cells after chronic treatment (6 months) with HIV-PIs.

Nevertheless, resistance for each of the HIV-PI selected cell lines may result from alterations in other ABC transporters, as some inhibitory activity of at least one HIV-PI has been shown for 11 family members of the ABC transporters (Bierman *et al*, 2010). Included in this list is ABCG2, an ABC transporter that has also been associated with MDR in certain cancers (Gottesman *et al*, 2002; Szakacs *et al*, 2006). Although we did not find this transporter significantly expressed at the protein level in SLK cell lines, the role of other drug transporters and/or other mechanism/s of drug resistance cannot be ruled out. These findings may be of particular interest as other common drugs employed in non-neoplastic diseases that are substrates for the ABC transporters have been reported to select for increased ABCB1 in tumour cells following *in vivo* treatment (Herzog *et al*, 1993) and to confer resistance to doxorubicin in a tumour system (Granzotto *et al*, 2004). Thus, it can be hypothesised that HIV-PIs may also exert their selection potential following *in vivo* administration. This may be of note as, beside AIDS-associated KS (Sgadari *et al*, 2002), HIV-PIs are under evaluation in a number of clinical trials as anticancer drugs for treatment of HIV-unrelated tumours, either alone (Brunner *et al*, 2008; Monini *et al*, 2009) or in association with known chemotherapeutic compounds (see http://www.clinicaltrials.gov).

Unlike the other tested HIV-PIs, indinavir requires significantly higher concentrations in order to select for an MDR phenotype (Tables 1 and 2). This may be of interest in light of the ongoing development of HIV-PIs as anticancer drugs, suggesting perhaps that some may be less likely to select for ABCB1 expression. However, 5 *μ*M of indinavir potentiates doxorubicin selection of ABCB1 and decreases chemosensitivity (Figure 1Ab and Tables 1 and 2). This synergistic effect is a common feature of all tested HIV-PIs and results from the overexpression in the plasma membrane of a functional ABCB1 protein. The overlapping of ABCB1 total protein, cell surface expression, and chemosensitivity hierarchies in co-selected SLK cell lines further supports this observation (Figures 1 and 2; Tables 1 and 2). Interestingly, the lopinavir/ritonavir combination shows the strongest synergistic effect when combined with doxorubicin, but when used without doxorubicin, this combination decreases ABCB1 protein expression, suggesting an antagonistic interaction between these two HIV-PIs (Figure 1). Moreover, these seemingly opposite results, also evident for other HIV-PIs, suggest that HIV-PIs could target different cellular pathways affecting different levels of ABCB1 regulation and/or the expression profiles of selected cells at the mRNA and protein levels. Although little is known about the signalling pathways targeted by the HIV-PIs in general, and how these pathways regulate ABCB1 in particular, previous reports found that nelfinavir treatment decreased signalling through the PI3K/Akt pathway (Gupta *et al*, 2007), a regulator of cellular proliferation and survival in human cancer cells including KS (Vivanco and Sawyers, 2002; Chaisuparat *et al*, 2008). Because of the fact that Akt upregulates ABCB1 (Garcia *et al*, 2009), further studies should determine whether the concentration-dependent alterations in ABCB1 expression by HIV-PIs are Akt dependent. Interestingly, HIV-PIs alter the nuclear hormone receptors PXR and SXR through induction of expression and enzyme activation, respectively (Zastre *et al*, 2009; Dussault *et al*, 2001), which can also induce ABCB1 expression (Callaghan *et al*, 2008). Furthermore, the fact that ATV1-selected cell lines express less ABCB1 mRNA than the parental but more protein (Figures 1Ad, B and C), suggests that HIV-PIs may be involved in the post-transcriptional regulation of ABCB1.

Although ABCB1 expression correlated with increased resistance to the HIV-PIs, doxorubicin and paclitaxel, any mechanism by which a drug's toxicity is undermined is a possible mechanism of resistance, including altered transport, metabolism, and drug targets. The aetiology of KS results in abnormal regulation of cell cycle, promotion of angiogenesis, and increased anti-apoptotic signalling, which could result in inherent resistance (Dittmer and Krown, 2007). Altered regulation (transcriptional, post transcriptional, and translational) and activity of metabolic enzymes could result in inefficient metabolism of drug. The HIV-PIs alter metabolic enzyme activity/expression for CYP3A5, CYP3A4, CYP2D6, and CYP2D16 *in vitro* and *in vivo* (Antoniou *et al*, 2005). Altered transportation of drug that prevents it from exerting its effects could result in MDR. For example, it has been suggested that increases in transporters at the nuclear membrane prevent doxorubicin from interacting with topoisomerase, its primary known target (Bao *et al*, 2011). Furthermore, organic anion transporters are known as uptake transporters for HIV-PIs, and therefore alterations in these transporters could result in lowered drug seen by a cell (Janneh *et al*, 2008). Overall, cancer is a dynamic process; each player could play a part in MDR.

In conclusion, our study shows that KS-derived cells exposed to HIV-PIs and doxorubicin develop an MDR phenotype that correlates with ABCB1 protein expression *in vitro*. Given the growing use of concomitant chemotherapy and combined antiretroviral therapy (cART) in HIV/AIDS (Mounier *et al*, 2009), further studies are needed to verify if this phenomenon occurs with other antiretroviral compounds that are substrates for the ABC transporters and other HIV-related and HIV-unrelated cancers *in vitro* and *in vivo* in patients administered life-long cART.

## Figures and Tables

**Table 1:** Growth inhibitory concentrations (IC_50_) and resistance factor (RF) to doxorubicin of parental and drug-selected SLK cell lines

**Treatment**	**Concentration (*μ*M)**	**IC_50_ (*μ*M)**	**IC_50_ (*μ*M) SLK parent**	**RF (IC_50_/IC_50_ SLK parent)**
Doxorubicin	0.02	1.42±0.32^*^	0.59±0.24	2.40
	0.04	2.76±0.6^*^	0.59±0.24	4.67
	0.09	3.72±0.56^*^	0.59±0.24	6.30
	0.2	7.4±1.01^*^	0.59±0.24	12.54
Indinavir	10	0.794±0.172	0.59±0.24	1.34
	30	1±0.166	0.59±0.24	1.69
	50	2.05±0.27^*^	0.59±0.24	3.46
Doxorubicin/indinavir	0.02/5	1.79±0.3^*^	0.59±0.24	3.02
Nelfinavir	2	1.21±0.30^*^	0.59±0.24	2.05
	5	1.74±0.33^*^	0.59±0.24	2.95
	10	0.98±0.32	0.59±0.24	1.66
Doxorubicin/nelfinavir	0.02/5	3.2±0.51^*^^†^	0.59±0.24	5.42
Atazanvir	1	0.43±0.04^*^	0.21±0.04	2.04
	5	0.93±0.14	0.59±0.24	1.57
	10	0.67±0.11	0.59±0.24	1.17
Doxorubicin/atazanvir	0.02/5	3.76±0.30^*^^†^	0.59±0.24	6.37
Ritonavir	1	0.26±0.022	0.21±0.04	1.23
	2.5	0.31±0.03^*^	0.21±0.04	1.47
	5	0.25±0.08	0.59±0.24	0.42
	10	0.54±0.18	0.59±0.24	0.91
	20	0.34±0.15	0.59±0.24	0.57
Doxorubicin/ritonavir	0.02/5	3.66±0.53^*^^†^	0.59±0.24	6.20
Lopinavir	10	0.72±0.14	0.59±0.24	1.22
Doxorubicin/lopinavir/ ritonavir	0.02/10/5	4.23±0.37^*^^†^	0.59±0.24	7.16

^*^P⩽0.05.

^†^Combination data were analyzed for synergism using the Bliss independence drug interaction model with a significance level of *P*⩽0.05.

**Table 2:** Growth inhibitory concentrations (IC_50_) and resistance factor (RF) to paclitaxel of parental and drug-selected SLK cell lines

**Treatment**	**Concentration of treatment (*μ*M)**	**IC_50_ (*μ*M) to paclitaxel**	**RF (IC_50_/IC_50_ SLK parent)**
None		0.2±0.008	
Doxorubicin	0.02	1.26±0.18^*^	6.3
	0.04	9.55±1.63^*^	47.75
Indinavir	10	0.11±0.04^*^	0.55
	50	1.7±0.12^*^	8.5
Doxorubicin/indinavir	0.02/5	2.12±0.49^*^	10.6
Nelfinavir	2	0.87±0.17^*^	4.35
	5	1.29±0.23^*^	6.45
	10	0.56±0.22^*^	2.8
Doxorubicin/nelfinavir	0.02/5	3.94±0.59^*^^†^	19.7
Atazanvir	10	0.41±0.08^*^	2.05
Doxorubicin/atazanvir	0.02/5	5.9±1.79^*^^†^	29.5
Ritonavir	5	0.44±0.11^*^	0.42
Doxorubicin/ritonavir	0.02/5	3.66±0.53^*^^†^	2.2
Lopinavir	10	0.33±0.05^*^	1.65
Doxorubicin/lopinavir/ ritonavir	0.02/10/5	243.73±45^*^^†^	1218.65

^*^P⩽0.05.

^†^Combination data were analyzed for synergism by using the Bliss independence drug interaction model with a significance level of *P*⩽0.05.
